# Factors Associated With Continuous Use of a Cancer Education Metaverse Platform: Mixed Methods Study

**DOI:** 10.2196/57762

**Published:** 2024-07-15

**Authors:** Sunghak Kim, Timothy Jung, Dae Kyung Sohn, Mina Suh, Yoon Jung Chang

**Affiliations:** 1 National Cancer Survivorship Center National Cancer Control Institute National Cancer Center Goyang Republic of Korea; 2 Faculty of Business and Law Manchester Metropolitan University Manchester United Kingdom; 3 Center for Colorectal Cancer National Cancer Center Goyang Republic of Korea; 4 Division of Cancer Early Detection National Cancer Control Institute National Cancer Center Goyang Republic of Korea

**Keywords:** metaverse, virtual reality, cancer education, cancer detection, digital health

## Abstract

**Background:**

Early detection of cancer and provision of appropriate treatment can increase the cancer cure rate and reduce cancer-related deaths. Early detection requires improving the cancer screening quality of each medical institution and enhancing the capabilities of health professionals through tailored education in each field. However, during the COVID-19 pandemic, regional disparities in educational infrastructure emerged, and educational accessibility was restricted. The demand for remote cancer education services to address these issues has increased, and in this study, we considered medical metaverses as a potential means of meeting these needs. In 2022, we used Metaverse Educational Center, developed for the virtual training of health professionals, to train radiologic technologists remotely in mammography positioning.

**Objective:**

This study aims to investigate the user experience of the Metaverse Educational Center subplatform and the factors associated with the intention for continuous use by focusing on cases of using the subplatform in a remote mammography positioning training project.

**Methods:**

We conducted a multicenter, cross-sectional survey between July and December 2022. We performed a descriptive analysis to examine the Metaverse Educational Center user experience and a logistic regression analysis to clarify factors closely related to the intention to use the subplatform continuously. In addition, a supplementary open-ended question was used to obtain feedback from users to improve Metaverse Educational Center.

**Results:**

Responses from 192 Korean participants (male participants: n=16, 8.3%; female participants: n=176, 91.7%) were analyzed. Most participants were satisfied with Metaverse Educational Center (178/192, 92.7%) and wanted to continue using the subplatform in the future (157/192, 81.8%). Less than half of the participants (85/192, 44.3%) had no difficulty in wearing the device. Logistic regression analysis results showed that intention for continuous use was associated with satisfaction (adjusted odds ratio 3.542, 95% CI 1.037-12.097; *P*=.04), immersion (adjusted odds ratio 2.803, 95% CI 1.201-6.539; *P*=.02), and no difficulty in wearing the device (adjusted odds ratio 2.020, 95% CI 1.004-4.062; *P*=.049). However, intention for continuous use was not associated with interest (adjusted odds ratio 0.736, 95% CI 0.303-1.789; *P*=.50) or perceived ease of use (adjusted odds ratio 1.284, 95% CI 0.614-2.685; *P*=.51). According to the qualitative feedback, Metaverse Educational Center was useful in cancer education, but the experience of wearing the device and the types and qualities of the content still need to be improved.

**Conclusions:**

Our results demonstrate the positive user experience of Metaverse Educational Center by focusing on cases of using the subplatform in a remote mammography positioning training project. Our results also suggest that improving users’ satisfaction and immersion and ensuring the lack of difficulty in wearing the device may enhance their intention for continuous use of the subplatform.

## Introduction

### Background

Breast cancer is one of the most common cancers affecting women. Survival rates can increase with early detection, which is linked to proper treatment [1,2]. Mammography is a widely accepted method for early detection of breast cancer that enables health professionals to detect tumors in patients with no signs or symptoms of the disease [3,4]. High-quality mammograms are essential for the successful early detection of breast cancer, and mammography positioning is one of the key elements in securing high-quality mammograms. Thus, mammography positioning training for radiologic technologists is crucial for improving the feasibility of successful early detection of breast cancer [5]. However, mammography education is challenging because of the lack of material resources [6]. One possible reason for the lack of material resources is that it is difficult to observe other people using mammography directly or to obtain realistic training resources. While other cancer screening education fields, such as endoscope cleaning and disinfection method training, are relatively easy to demonstrate externally, mammography positioning training is relatively difficult to demonstrate externally because of its association with personal privacy issues and sensitivity. In addition, the outbreak of infectious diseases such as COVID-19 and the disparity in cancer screening and diagnosis educational systems among medical institutions have led to a decline in access to excellent cancer education [7,8]. Overcoming these problems requires efficient digital health care services that enable comprehensive, systematic, and continuous remote cancer education. In particular, producing material resources for mammography positioning training using metaverse technology may help alleviate the challenges of the lack of material resources in the real world. Therefore, this study selected remote mammography positioning training in the metaverse subplatform as its research topic.

National Cancer Center (NCC) Korea’s Division of Cancer Early Detection runs a national cancer screening support project aimed at increasing the cure rate and reducing cancer-related mortality by inducing early cancer detection and treatment. The division leads regionally driven quality improvement activities of national cancer screening to enhance its accuracy and reliability and to perform regular training in each region to reduce regional disparities in the quality of national cancer screening [9,10]. The division provides various remote educational programs and content for training purposes, including non–face-to-face virtual reality (VR) educational videos for mammography positioning training. However, the previous way of providing VR educational videos to each regional cancer center (RCC) required significant manual effort during the delivery process. The division recognized the need for a digital platform to facilitate content delivery and management and continuous non–face-to-face education.

The need for a digital platform for the early detection of cancer aligned well with the Dr. Meta metaverse platform’s need for content variety. We developed a multipurpose metaverse digital cancer care platform, Dr. Meta, at NCC Korea in 2021, supported by the Republic of Korea’s Ministry of Science and ICT [11]. The Dr. Meta metaverse platform showed potential for successful cancer control; however, according to participants’ feedback, its weakness was the lack of content diversity [11]. In 2022, supported by the Republic of Korea’s Ministry of Health and Welfare, we upgraded the metaverse platform to improve the Dr. Meta user experience by embedding new educational VR content in the Metaverse Educational Center subplatform and adding a new VR Healing Theater subplatform under the Dr. Meta platform [12]. Especially, the Metaverse Educational Center subplatform, which helps users overcome physical constraints (eg, time and space) in education and conveniently receive training on demand, was developed to actively respond to the demand for non–face-to-face services in the post–COVID-19 era. The subplatform is equipped with multimedia so that users can upload and interact with various medical training materials, ranging from typical documents, images, videos, presentation slides, and webpages to 3D object data and VR content created using 360° video technology. In this interactive environment, users can directly control and interact with the subplatform’s interface, virtual objects, and spatial elements and be more immersed with the subplatform. Users can also look in any direction while playing the educational VR content and have immersive experiences in this subplatform [11,12]. As the previous studies show that immersion and interaction are associated with the intention to continuously use the VR services, it is anticipated that such improved immersion may positively affect the intention for continuous use of the subplatform [13,14]. However, improving the diversity of content in the subplatform was a challenge [11]. Consequently, we collaborated with the Division of Cancer Early Detection of NCC Korea in 2022 using Metaverse Educational Center to virtually educate radiologic technologists working at each RCC on mammography positioning.

Previous studies have shown that the metaverse can serve as a promising tool in cancer education [15-17]. However, to effectively use the tool, it is insufficient to merely know the positive relationship between the metaverse and cancer education. Identifying the factors that may affect the use of metaverse services is important for creating successful strategies for developing, upgrading, and operating them and maximizing their effectiveness. Numerous studies have explored possible factors that can impact the use of metaverse technology, frequently applying the technology acceptance model (TAM) to identify meaningful factors [18-20]. On the basis of the TAM, perceived usefulness and perceived ease of use of technology impact satisfaction and, subsequently, intention to use [21]. In this study, we considered TAM-based factors when developing a metaverse platform and evaluating the user experience. In the context of remote mammography positioning training for radiologic technologists, this study goes beyond the simple usability test of Metaverse Educational Center and examines the factors and relationships associated with using the subplatform for an advanced understanding of its use.

### Study Objectives

This study investigated the user experience of the Metaverse Educational Center subplatform and the factors associated with the intention for continuous use by focusing on cases of using the subplatform in a remote mammography positioning training project. We believe that Metaverse Educational Center is an outstanding digital tool for cancer education. As there are other educational VR contents being produced for home-based, hospice, non–face-to-face practice training and endoscope cleaning and disinfection method training in the Metaverse Educational Center subplatform, this study can serve as evidence of this subplatform’s wide applicability across various training topics and target groups if positive outcomes are found. Furthermore, we expected that study outcomes suggest practical strategies for improving the quality of Metaverse Educational Center and facilitating its successful dissemination and implementation.

## Methods

### Study Design

After demonstrating the Metaverse Educational Center subplatform of the Dr. Meta metaverse platform to participants, we conducted a multicenter, cross-sectional survey to examine their experiences with the subplatform. Quantitative data were collected through Likert-scale survey questions, and qualitative data were collected through an open-ended question.

### Procedure

In 2022, we disseminated Dr. Meta to 12 RCCs (ie, Ajou University Hospital, Chonnam National University Hwasun Hospital, Chungbuk National University Hospital, Chungnam National University Hospital, Gachon University Gil Medical Center, Gyeongsang National University Hospital, Jeju National University Hospital, Jeonbuk National University Hospital, Kangwon National University Hospital, Kyungpook National University Chilgok Hospital, Pusan National University Hospital, and Ulsan University Hospital). On the basis of goal of positively contributing to the efficiency of national cancer screening through VR education, we collaborated with NCC Korea’s Division of Cancer Early Detection and installed the Division of Cancer Early Detection VR educational videos for mammography positioning training within our Metaverse Educational Center subplatform. After preparing for the launch of the remote mammography positioning training program, we conducted virtual sessions for radiologic technologists through the Metaverse Educational Center subplatform in cooperation with the Division of Cancer Early Detection. The survey was conducted after the completion of virtual sessions.

### Study Participants

**Figure 1:**
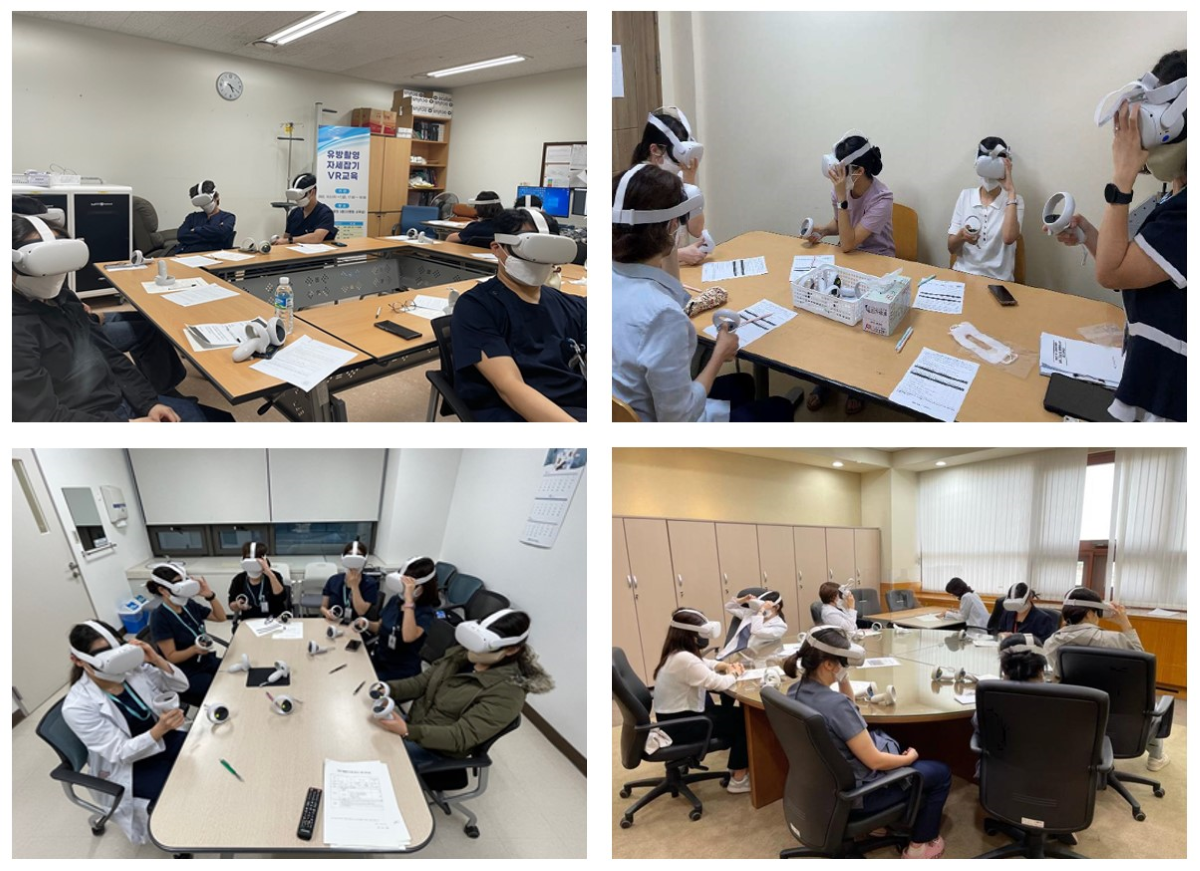
Pictures of participants using Metaverse Educational Center.

Metaverse Educational Center usability tests were conducted at 13 cancer centers (the NCC and 12 RCCs). Participants aged >19 years were recruited from each cancer center between July and December 2022. Inclusion criteria for the study were having experience in using Metaverse Educational Center and being aged >19 years. Exclusion criteria for the study were having experience in using the subplatform but being aged ≤19 years, having a severe physical or mental condition that prevented them from participating in the study, being unable to complete the self-reported survey, not consenting to the survey, or withdrawing consent during the study. The remote mammography positioning training program was designed to educate radiologic technologists, so most study participants were radiologic technologists. Doctors, nurses, and hospital administrative staff also participated in this study. Figure 1 shows images of participants using Metaverse Educational Center.

### Ethical Considerations

The study was approved by the institutional review board of NCC Korea (IRB: NCC2022-0209). Only those who read the research information and voluntarily consented to participate in the survey were able to participate. Data used in this study were anonymous because participants were not asked for identifying information (eg, name and social security number). Users who participated in the study received an everyday item worth approximately ₩7000 (US $5) as a reward.

### Measures

In the survey preparation phase, we asked platform users about their impressions of the initially prepared questionnaire. Their responses conveyed a preference to mitigate perceived burdens when participating in the survey after having professional virtual training through new metaverse technology. Therefore, we used theory-based but easy and short survey questions to facilitate participants’ understanding of the questions among diverse participant groups. The survey questions were adapted from previous studies examining the TAM in the context of metaverse services [11,12,22], translated into Korean, abbreviated, and modified to suit the purpose and context of the study. In addition, we adopted the viewpoint of separating the experience of technology use into hardware and software aspects [22,23]. Researchers with expertise in communication, metaverse, and health translated survey questions from English into Korean, followed by consensus to determine the most suitable translation. Subsequently, the questionnaire was reviewed by an expert proficient in both Korean and English, who was separate from the research team, to complete the final version. First, participants were asked to provide their sociodemographic information (age, sex, and position in the hospital). Next, they were asked to assess their individual differences (2 items: “I am usually interested in using new technologies or devices” and “I tend to not experience dizziness or motion sickness easily”) and user experiences (8 items: “I was generally satisfied with using this subplatform,” “This subplatform made me feel interested,” “This subplatform made me feel immersed,” “This subplatform was easy to use,” “There was no discomfort (eg, dizziness and nausea) in using this subplatform,” “There was no difficulty in wearing this subplatform device (ie, head-mounted display and controllers),” “There was no difficulty in operating this subplatform device (ie, head-mounted display and controllers),” and “I want to continue using this subplatform in the future”) relevant to the use of Metaverse Educational Center. Each item was scored on a 4-point Likert scale (1=“strongly disagree” to 4=“strongly agree”). Finally, participants were asked to provide their feedback to improve Metaverse Educational Center by answering the open-ended question (“If you have any suggestions or improvements, please write them in”). We used these 10 quantitative questions and the open-ended qualitative question to evaluate participants’ experiences with Metaverse Educational Center*.* The questionnaire used in this study is presented in Multimedia Appendix 1.

### Statistical Analysis

First, we conducted a descriptive analysis to examine how participants perceived their experiences using Metaverse Educational Center. The user experience was rated by obtaining the frequency and proportion of participants with positive opinions for each survey question. The number of participants with positive opinions after using Metaverse Educational Center was calculated by adding those who answered “agree” and “strongly agree.” Next, we performed a logistic regression analysis to identify the variables associated with the intention to continue using the subplatform. To create a binary dependent variable, we categorized participants’ reaction values to a survey question relevant to intention for continuous use into 2 groups (“strongly disagree”+“disagree” vs “agree”+“strongly agree”). Univariable logistic regression analyses were performed for each variable to obtain odds ratios with 95% CIs of intention for continuous use. A multiple logistic regression analysis was performed to identify factors related to this dichotomous dependent variable using only variables with statistical significance from the univariable logistic regression analyses. Finally, we qualitatively reviewed the feedback acquired from the open-ended question on the improvement of the subplatform. To identify and interpret the main themes and patterns within the feedback data with the researchers’ subjectivity, we conducted a reflexive thematic analysis by following the 6 phases of analysis (familiarizing yourself with the data set; coding; generating initial themes; developing and reviewing themes; refining, defining, and naming themes; and writing up) [24-26]. We read and reread the data to become familiar with its content. After that, we captured important data content and features relevant to the user experience of the subplatform and generated them as several codes. On the basis of collated codes, we generated and refined each theme in a recursive process and finalized its name to tell the best story of the data. We developed such a story into a discussion of the advantages and improvements of the subplatform and suggested future directions for upgrading the subplatform. The research team has multidisciplinary academic backgrounds in communication, metaverse, cancer prevention, cancer screening, and cancer treatment and has various experiences conducting digital health research. This researcher reflexivity may have influenced data analysis. In addition, we applied a content analysis approach to quantify the qualitative feedback data and assist the discussion derived from the reflexive thematic analysis. Data analysis was conducted using IBM SPSS Statistics V22.0 (IBM Corp).

## Results

### Participant Characteristics

Overall, 196 individuals participated in the survey after using the Metaverse Educational Center subplatform, and data from 192 participants were analyzed. Among the 192 participants, 74 (38.5%), 69 (35.9%), 38 (19.8%), and 11 (5.7%) were aged 20 to 29, 30 to 39, 40 to 49, and 50 to 59 years, respectively. In addition, 16 (8.3%) participants were male, and 176 (91.7%) were female. Regarding position in the hospital, most participants (160/192, 83.3%) fell into the “Health professionals (other)” category. These results can be attributed to mammography positioning training for radiologic technologists using Metaverse Educational Center in collaboration with the Division of Cancer Early Detection of NCC Korea in 2022. Table 1 presents participants’ demographic characteristics.

**Table 1:** Demographic information of participants (N=192).

Characteristics			Participants, n (%)^a^
**Age group (y)**			
	20-29	74 (38.5)	
	30-39	69 (35.9)	
	40-49	38 (19.8)	
	50-59	11 (5.7)	
**Sex**			
	Male	16 (8.3)	
	Female	176 (91.7)	
**Position in the hospital**			
	Health professionals (doctors)	10 (5.2)	
	Health professionals (nurses)	19 (9.9)	
	Health professionals (other)^b^	160 (83.3)	
	Other^c^	3 (1.6)	

^a^Percentages may not add up to 100% due to rounding.

^b^“Health professionals (other)” includes radiologic technologists, physical therapists, and pharmacists in this study.

^c^“Other” includes hospital administrative staff, educators, social workers, and researchers in this study.

### Participants’ Experiences Using the Metaverse Educational Center Subplatform

We conducted a quantitative descriptive analysis to examine how participants perceived their experiences using Metaverse Educational Center. Our results showed that most participants (178/192, 92.7%) were satisfied with their user experience of Metaverse Educational Center. Most participants responded that Metaverse Educational Center was an interesting (184/192, 95.8%) and immersive (174/192, 90.6%) subplatform. A total of 138 (71.9%) participants reported that the subplatform was easy to use. Most participants (157/192, 81.8%) wanted to continue using the subplatform in the future. Table 2 presents the overall positive experiences of Metaverse Educational Center’s participants.

**Table 2:** Participants’ positive experiences after using Metaverse Educational Center (N=192).

Items^a^			Participants, n (%)^b^
**Individual differences**			
	I am usually interested in using new technologies or devices.	167 (87)	
	I tend to not experience dizziness or motion sickness easily.	99 (51.6)	
**User experiences**			
	I was generally satisfied with using this subplatform.	178 (92.7)	
	This subplatform made me feel interested.	184 (95.8)	
	This subplatform made me feel immersed.	174 (90.6)	
	This subplatform was easy to use.	138 (71.9)	
	There was no discomfort (eg, dizziness and nausea) in using this subplatform.	103 (53.6)	
	There was no difficulty in wearing this subplatform device (ie, head-mounted display and controllers).	85 (44.3)	
	There was no difficulty in operating this subplatform device (ie, head-mounted display and controllers).	110 (57.3)	
	I want to continue using this subplatform in the future.	157 (81.8)	

^a^Items are based on a 4-point scale (ranging from 1=“strongly disagree” to 4=“strongly agree”). The number of participants who had positive opinions after using Metaverse Educational Center was calculated by adding the number of participants who answered “agree” and “strongly agree.”

^b^Percentages may not add up to 100% due to rounding.

### Factors Associated With Intention for Continuous Use of the Metaverse Educational Center Subplatform

We conducted a quantitative logistic regression analysis to identify the variables associated with the intention to continue using the subplatform. In univariable logistic regression analyses, a usual interest in using new technologies or devices (odds ratio 1.998, 95% CI 1.141-3.497; *P*=.02), satisfaction (odds ratio 8.079, 95% CI 3.284-19.875; *P*<.001), interest (odds ratio 3.000, 95% CI 1.570-5.731; *P*=.001), immersion (odds ratio 5.753, 95% CI 2.985-11.088; *P*<.001), perceived ease of use (odds ratio 2.973, 95% CI 1.741-5.077; *P*<.001), no discomfort in use (odds ratio 2.021, 95% CI 1.287-3.173; *P*=.002), and no difficulty in wearing the device (odds ratio 2.494, 95% CI 1.448-4.296; *P*=.001) were statistically different between participants not having intention for continuous use and participants having intention for continuous use. In the multiple logistic regression analysis, participants who were generally satisfied with using Metaverse Educational Center were more likely to intend to continue use the subplatform than those who were not (adjusted odds ratio 3.542, 95% CI 1.037-12.097; *P*=.04). When immersion increased by one unit, users were 2.803 times more likely to have intention for continuous use (adjusted odds ratio 2.803, 95% CI 1.201-6.539; *P*=.02). Having no difficulty in wearing the device was positively associated with intention for continuous use (adjusted odds ratio 2.020, 95% CI 1.004-4.062; *P*=.049). Table 3 presents the results of the univariable and multiple logistic regression analyses for each variable.

**Table 3:** Factors associated with intention for continuous use of Metaverse Educational Center.

Items		Intention for continuous use			
		Univariable		Multiple	
		Odds ratio (95% CI)	*P* value	Adjusted odds ratio (95% CI)	*P* value
**Sociodemographics**					
	Age group (y)	0.976 (0.651-1.466)	.91	—^a^	—
	Sex	1.559 (0.471-5.157)	.47	—	—
	Position in the hospital	1.741 (0.980-3.093)	.06	—	—
**Individual differences**					
	I am usually interested in using new technologies or devices.	1.998 (1.141-3.497)	.02	1.823 (0.949-3.502)	.07
	I tend to not experience dizziness or motion sickness easily.	1.282 (0.833-1.973)	.26	—	—
**User experiences**					
	I was generally satisfied with using this subplatform.	8.079 (3.284-19.875)	<.001	3.542 (1.037-12.097)	.04
	This subplatform made me feel interested.	3.000 (1.570-5.731)	.001	0.736 (0.303-1.789)	.50
	This subplatform made me feel immersed.	5.753 (2.985-11.088)	<.001	2.803 (1.201-6.539)	.02
	This subplatform was easy to use.	2.973 (1.741-5.077)	<.001	1.284 (0.614-2.685)	.51
	There was no discomfort (eg, dizziness and nausea) in using this subplatform.	2.021 (1.287-3.173)	.002	1.212 (0.682-2.153)	.51
	There was no difficulty in wearing this subplatform device (ie, head-mounted display and controllers).	2.494 (1.448-4.296)	.001	2.020 (1.004-4.062)	.049
	There was no difficulty in operating this subplatform device (ie, head-mounted display and controllers).	1.475 (0.928-2.346)	.10	—	—

^a^Not applicable.

### Feedback on the Metaverse Educational Center Subplatform

We conducted a qualitative feedback review to explore participants’ diverse opinions regarding the use of the subplatform. Participants answered the open-ended question “If you have any suggestions or improvements, please write them in.” We broke down the 145 participants’ responses at the sentence-unit level and collected 276 sentences. We then sorted these sentences into groups with homogeneous attributes and labeled them accordingly. Participants provided a wide range of feedback on the advantages and improvements of Metaverse Educational Center. Through the reflexive thematic analysis, we identified several important themes related to the subplatform’s advantages (eg, “immersion and concentration,” “usefulness,” and “satisfaction”) and understood how they are manifested in the data. We also identified various themes related to the subplatform’s improvements in hardware (eg, “heaviness of the device,” “difficulties in wearing the device,” and “difficulties in operating the device”) and software (eg, “deficiency of interactivity in the content,” “deficiency of content variety,” and “poor qualities of the images”) aspects and understood how they relate to one another. In addition, the frequency of each theme was calculated by applying the content analysis approach to complement the outcomes of the reflexive thematic analysis. Regarding the subplatform’s advantages (21 sentences), the most commonly reported label was “usefulness.” Regarding the subplatform’s improvements (255 sentences), the most commonly reported one was “deficiency of interactivity in the content.” “Deficiency of content variety” and “heaviness of the device” were other representative improvements mentioned by participants. Table 4 presents more information about the feedback on the participants’ experiences with Metaverse Educational Center.

**Table 4:** Feedback details of participants’ experiences of Metaverse Educational Center.

Labels		Examples	Sentences, n (%)^a^
**Advantages (n=21)**			
	No inconvenience	“There was no inconvenience in using Metaverse Educational Center because I was well-instructed on how to wear and operate the device.”	2 (9.5)
	Immersion and concentration	“This training method is likely effective because it raises immersion and concentration.”	2 (9.5)
	Usefulness	“It was good to obtain in-depth knowledge through this educational metaverse platform.” “If a few more improvements are made, this subplatform can be used for educational purposes in more areas.”	6 (28.6)
	Ease of understanding	“The explanations of the topics and content of the training were easy to understand.”	2 (9.5)
	Vividness and sense of presence	“The educational materials were vividly delivered and made me feel like I was there.”	4 (19)
	Interest	“Unlike viewing the educational materials in a fixed position on a personal computer, I could move and interact with them, so it was fun and not boring.”	1 (4.8)
	Satisfaction	“Overall, I was satisfied with the experience.”	4 (19)
**Improvements (n=255)**			
	Difficulties in using the software	“I wish this subplatform changed to be more accessible and more straightforward.” “It was difficult to operate the cancer education metaverse platform, and there were too many options that made me confused.”	10 (3.9)
	Poor settings of the screen focus	“It was difficult to freely set the screen’s focus, angle, and height in the direction I wanted or as if I was looking from the front.” “I was forced to move my head or body continuously while watching the screen as its perspective was not smoothly switched, and this experience was a bit uncomfortable and painful.”	26 (10.2)
	Poor qualities of the images	“The qualities of images need to be improved.” “I would like to see educational materials with more stereoscopic and clearer visuals.”	27 (10.6)
	Dizziness, fatigue, and distraction	“I felt dizzy and had eye strain at the end of the training.” “I could not concentrate well in the middle of watching the video.”	19 (7.5)
	Issues of noise	“I could not pay attention to the training because this educational platform was vulnerable to external noise, and reversely, the platform sounds echoing outward made me realize the need for earphones or a headset.”	7 (2.7)
	Lengthy videos	“The video was too long, preventing me from adequately closing my eyes or taking a break while watching.”	2 (0.8)
	Poor internet connections	“I experienced freezing while playing videos or navigating the platform space.”	2 (0.8)
	Difficulties in wearing the device	“Wearing the device of this subplatform was frustrating and uncomfortable for me because the device was pressing my head and nose and its fit was not seamless.” “The device of this subplatform did not fit properly and was too loose and fogged up.”	24 (9.4)
	Heaviness of the device	“The device of this subplatform was heavy.” “As Metaverse Educational Center’s device was heavy, I would like to see improvements in making it lighter or adding instruments that support wearing it stably.” “Metaverse Educational Center’s device was heavy and uncomfortable to wear for long periods.”	33 (12.9)
	Difficulties in operating the device	“Operating the device of this subplatform was too complicated and needed to be simplified.” “Sometimes, errors occurred during the operation of this subplatform’s device, hindering the smooth progress of training.”	20 (7.8)
	Physical constraints	“It was uncomfortable to be restricted to the particular location and equipment requirements to experience this service.”	1 (0.4)
	Deficiency of content variety	“I want to get training through this educational subplatform not only for mammography positioning but also for other topics.” “I hope different mammography positioning cases and methods for different patients will be handled on educational materials as each patient has different characteristics.” “I would like to see cancer education programs related not only to screening but also to treatment.”	34 (13.3)
	Deficiency of interactivity in the content	“I want to control and interact more with the digital content of this subplatform, not just watch it.” “It would be great to have a realistic participatory simulation feature in this subplatform so users can practice mammography positioning.” “As the educational program is created based on metaverse technologies, seeing more immersive and engaging content that allows users to amplify the advantages and uniqueness of metaverse by manipulating the device would be nice.”	42 (16.5)
	Requests for function enhancement	“I want this subplatform to work both online and offline, and I want its virtual environment to be closer to the real world along with containing more controlling functions.”	7 (2.7)
	Necessity of different training methods	“It would be nice to have other training methods in case people have difficulties using Metaverse Educational Center, as it is different for everyone to feel motion sickness after using this educational metaverse platform.”	1 (0.4)

^a^Percentages may not add up to 100% due to rounding.

## Discussion

### Principal Findings

This study investigated the user experience of the Metaverse Educational Center subplatform and the factors associated with intention for continuous use by focusing on cases of using the subplatform in a remote mammography positioning training project. As securing a sufficient number of participants was difficult in the first study using the Dr. Meta metaverse platform [11], we were able to examine this metaverse platform’s user experience but were unable to reveal more detailed relationships among these factors. However, in this study, we secured more participants than in the first study of Dr. Meta use [11] by inviting new RCCs to our project and recruiting participants for an extended period. We also installed specific purpose-driven VR content produced for use in an actual cancer education project in cooperation with the Division of Cancer Early Detection of NCC Korea. On the basis of the increased sample size and new metaverse cancer education content, this study targeted the subplatform in an obvious context, complemented the methodology, and addressed its user experience in various ways.

Our results, focusing on cases of using Metaverse Educational Center in a remote mammography positioning training project, showed the positive user experience of the subplatform. Most users considered the subplatform satisfactory, interesting, and immersive. More than half of the users considered the subplatform easy to use. Although less than half of the users indicated positive opinions regarding the lack of difficulty in wearing this subplatform device, more than half of the users expressed positive opinions on the lack of discomfort in using the subplatform and lack of difficulty in operating its device. Finally, most users wanted to use the subplatform continuously. The positive user experience of the subplatform indicates that it can also be successfully used in another project of the Division of Cancer Early Detection (endoscope cleaning and disinfection method training). We intend to enhance the scalability of the subplatform and its use in the future by developing metaverse content covering various cancer education topics and mounting them into the subplatform.

Our study’s outcomes demonstrated factors related to the intention to continue using Metaverse Educational Center. According to the logistic regression analysis results, the associations between intention for continuous use of Metaverse Educational Center and satisfaction, immersion, and lack of difficulty in wearing the device were statistically significant. In previous studies, satisfaction, immersion, and lack of difficulty in wearing the device have been identified as factors directly or indirectly related to the intention to continuously use metaverse or other interactive media services [13,27,28]. Our results are consistent with those of previous studies. A higher level of satisfaction may increase the likelihood of intention for continuous use [22,27]. Users immersed in metaverse content may lose their self-consciousness, concentrate intensely on the content, and become satisfied with it [13,29]. Users may pursue an easy and relaxed experience of wearing the device, and, therefore, they are more likely to use the device if it is not difficult to wear continuously [28,30,31].

Conversely, the associations between the intention for continuous use of Metaverse Educational Center and interest, perceived ease of use, and lack of discomfort in use were not statistically significant. No associations of intention for continuous use with interest, perceived ease of use, or discomfort in use were unexpected because their associations (whether direct or indirect) have often been observed in previous studies [32-34]. These discrepancies may be due to different contextual characteristics. This study provided remote mammography positioning training to health professionals. Therefore, users may have had a strong motivation to learn skills, and the content may have been produced to deliver professional knowledge as a top priority. Entertainment, ease of use, and comfort may have weak relevance to the strong purposefulness of training; interest, perceived ease of use, and lack of discomfort in use may not be associated with the intention for continuous use of the educational metaverse platform. In addition, many younger individuals known to be familiar with the metaverse [35] and many radiologic technologists known to be familiar with complex medical devices [36] participated in this study. We assume that they tend to use emerging technologies to accomplish goals frequently; that is, they have high technology readiness [37]. However, contrary to the expectation of positive associations [38,39], neither age group nor position in the hospital showed a significant association with the intention for continuous use of the Metaverse Educational Center subplatform. Although younger participants may be familiar with metaverse services for purposes such as entertainment, they may not have been familiar with metaverse services for medical training. Furthermore, radiologic technologists may be familiar with the medical devices they usually handle but may be unfamiliar with metaverse devices, which can be considered relatively cutting-edge and perceived differently from other medical devices. This discussion implies that scrutinizing external variables such as the characteristics of the target period, target population group, and other situational contexts is vital when exploring factors associated with the intention for continuous use of metaverse services, including the Metaverse Educational Center subplatform. Various media theories, including the TAM, have evolved to include external variables for better explanatory power [40]. Therefore, subsequent research is needed to delve into the mixed results regarding the associations of intention for continuous use contingent on external variables to understand the reasons behind them.

### Implications

Discovering the factors associated with the intention to continuously use the Metaverse Educational Center subplatform has both theoretical and practical implications. With regard to theoretical implications, knowledge of these statistically supported associations may trigger research into the mechanisms or conditions of associations. Applying mixed methods approaches directly ask participants deeper about quantitative results regarding which factors are associated, in what direction, and why it can be one of the future research directions. Such attempts to understand the patterns of association may lead to the development of a systematic theoretical framework. Regarding practical implications, information about factors related to the intention for continuous use of the subplatform can guide the development of more effective intervention strategies that encourage its continuous use. It will be feasible to save time and cost for upgrading the subplatform and promoting its user experience by prioritizing factors that are likely to affect intention for continuous use.

Furthermore, the feedback on Metaverse Educational Center–shaped discourse pointed to future directions for upgrading the subplatform. Regarding the subplatform’s advantages, the total number of relevant sentences was smaller than that of sentences related to the subplatform’s improvements because the question did not directly ask about the advantages. However, the usefulness of the subplatform was remarkable. As the usefulness of the Metaverse Educational Center subplatform is proven, it will be possible to increase the quantity and quality of educational content helpful in gaining knowledge with varying topics and formats and expand the range of use of the subplatform. Such upgrading strategies can facilitate dynamic virtual training and activate the use of the subplatform among various user groups. Regarding the subplatform’s improvements, the deficiency of interactivity in the content, deficiency of content variety, and heaviness of the device were the main weaknesses. Poor screen focus and image quality, which lead to negative visual experiences, were also notable weaknesses. Despite upgrading the Dr. Meta metaverse platform based on the results of the first study to use the platform [11], the results of this study indicate that further quantitative and qualitative enhancements are needed for the content and devices of the subplatform. To improve the usability of the subplatform, it is crucial to address these weaknesses by securing diverse interactive metaverse content, solving the device’s weight problem, and improving functions related to screen focus control and image quality.

In addition, the feedback substantially supported the results of the usability test. The outcomes of the descriptive and logistic regression analyses were similar to those of the responses to the feedback question. More than half of the users thought that the Metaverse Educational Center subplatform was satisfactory, interesting, immersive, and easy to use. Less than half of the users believed that there was no difficulty in wearing the subplatform device. The former was addressed in the feedback as an advantage, whereas the latter was mentioned as an improvement. Factors associated with the intention for continuous use of the subplatform (ie, satisfaction, immersion, and lack of difficulty in wearing the device) also appeared in the feedback. Beyond emphasizing quantitative results, the feedback also clarifies new elements that received little attention but might be related to the user experience with the subplatform. For example, improving the audio system, reducing the runtime of content, and optimizing the operation of content would attract users’ motivation to experience the subplatform.

The feedback also suggests that certain advantages and improvements may be closely related. For example, some participants found it easier to understand the educational content through immersion (eg, “The content was easily understandable due to high immersion” [immersion and concentration - ease of understanding]), and some were overall satisfied with the subplatform because the educational content was beneficial to them (eg, “I was satisfied with this subplatform because I could get helpful information” [usefulness - satisfaction]). Some participants complained of dizziness, fatigue, and distraction due to the lack of breaks, lengthy videos, and blurry image quality when receiving training through the subplatform (eg, “The educational video was too long, so it was unable to rest for a long time. Even the image quality was not sharp. I was dizzy, tired, and had decreased concentration after finishing the training” [poor qualities of the images; lengthy videos - dizziness, fatigue, and distraction]). Some also expressed that wearing the device was challenging due to its heavy weight (eg, “The device was heavy and easy to slip off, making it difficult to wear” [heaviness of the device - difficulties in wearing the device]). Some connected the difficulties in wearing the device with wearing glasses (eg, “It was hard to wear the device to the extent that I had to take off my glasses during the education session. To make matters worse, the poor quality of the educational content’s image disrupted my learning” [difficulties in wearing the device; there was no specific categorized label about wearing glasses]). Such feedback can serve as indirect evidence for inferring new potential factors, their relationships, and directions. Determining whether these ideas are statistically valid could also be a topic for future research. By probing the feedback, we could reconfirm the results obtained from statistical methods and deal with more profound levels of user experience statements that are seldom obtained from quantitative approaches.

### Future Perspective

This study used a mixed methods design to comprehensively understand the usability of the Metaverse Educational Center subplatform while embracing diverse user experiences. Through quantitative approaches, this study suggests that the upgrade direction of improving factors associated with the intention for continuous use of the subplatform, that is, immersion and lack of difficulty in wearing the device, may effectively enhance the user experience and lead to future successful effects. In addition, through qualitative approaches, this study identified ideas for materializing this upgrade direction.

First, enhancing users’ perceived interactivity is an option to increase immersion. Studies have shown that perceived interactivity of new media, including metaverse and other interactive media, is positively associated with immersion [41-43]. In this study, although most participants thought that the subplatform was immersive, there was also considerable feedback indicating that participants wished for the educational content to be more interactive. Adding content scenarios and features that allow users to directly manipulate the 3D equipment needed for mammography or correct patients’ mammography positioning in a virtual environment through the switch buttons on the subplatform device would help increase perceived interactivity. The incorporation of participatory content that induces user engagement and sensory expansion through hands-on practice in a virtual space after theoretical education would also be beneficial. By improving interactive content in which users can directly control virtual spaces and digital objects relevant to mammography positioning training, it is possible to enhance the immersion of the subplatform beyond its current level and sustainably increase intention for continuous use.

Next, reducing the device weight is an option to decrease the difficulty in wearing the device. Previous studies have asserted that the heavy weight of a head-mounted display makes it difficult to wear and serves as a barrier preventing the activation of VR service [28,31]. In this study, less than half of the participants thought there was no difficulty in wearing the subplatform device, and there was considerable feedback claiming difficulties in wearing the device. Some feedback indicated that it was uncomfortable to wear the device because of its heavy weight, allowing us to infer the relationship between difficulties in wearing the device and its heaviness. Other feedback also mentioned that the subplatform device pressed on the nose or placed strain on the neck with fatigue, which seemed to be caused indirectly by the heaviness of the device. The difficulties in wearing the device and its heaviness issues were already acknowledged in the first Dr. Meta study [11]. Some improvements had been made using accessories to lighten the device weight; however, the results of this study revealed that further improvements are needed. If the difficulties in wearing the device cannot be adequately solved by improving the head-mounted displays, accessories, or peripheral devices, replacing them with entirely new devices could also be an option. If the disadvantages of a head-mounted display, such as difficulties in wearing it [28], outweigh its advantages, such as greater immersion by blocking additional visual inputs and other external stimuli [44], it may be more efficient to mitigate the disadvantages, even at the expense of some advantages. Overall engagement may slightly decrease if the subplatform is experienced on existing devices, such as desktops, laptops, cellphones, or tablets. However, the engagement inherent in the characteristics of metaverse content will be maintained, and difficulties in wearing the device will largely disappear. Most importantly, if users are given the choice of device to use for the subplatform by themselves, they can weigh the pros and cons and select it according to their needs and preferences. As a self-tailored experience, this may increase user satisfaction [45]. As difficulties in wearing the device may impair immersion [28], it would be beneficial to develop web and app modes and compatible modes to allow users to use the subplatform on familiar or mobile devices. Development of a new use model that can be run on different portable devices can alleviate difficulties in wearing the device, improve accessibility to the educational content, and complement the existing use model in diffusing the subplatform.

In this study, although not addressed by quantitative approaches, we obtained specific feedback related to the deficiency of content variety, following the first Dr. Meta study [11]. Participants wanted to receive not only mammography positioning training but also other mammography-related education through the Metaverse Educational Center subplatform and showed interest in cancer education on different topics; meeting these demands requires diverse scenarios, digital objects, and virtual environments. Applying artificial intelligence (AI) technology can effectively solve deficiencies in content variety. Scholars argue that embedding generative AI that creates images and 3D digital objects into the metaverse can save the time, energy, and costs associated with content development [46,47]. Moreover, using AI to collect and analyze metaverse use data can further advance service automation and personalization [48,49]. If AI combined with a metaverse captures and analyzes user experience and choice data, it can dynamically change virtual backgrounds and digital objects in real time to match user needs. Nonetheless, many hurdles remain in successfully integrating the metaverse and AI. The controversy related to copyright issues may limit the use of AI for content development in the metaverse. Legal deliberations and discussions are imperative to determine the ownership of copyrights concerning medical education content generated by AI, whether they reside in the final product or the source materials used for AI training, alongside their respective proportions. Data privacy violation is also a potential challenge for using AI to collect and analyze metaverse use data. Defining the scope of data use and establishing data protection methods are indispensable throughout the data collection, processing, and application phases [49]. It is essential to establish policies and ethics related to the use of metaverses and AI, and data security must be ensured. If these prerequisites are met, the industry will rapidly accelerate toward building data-driven metaverse systems using AI in the future. A successful combination of metaverses and AI may also appeal to participants to continuously use the subplatform.

Finally, the positive study outcomes in the user experience of the Metaverse Educational Center subplatform prove the extension of using this subplatform widely in not only various training topics and target groups but also different geographical locations. At the beginning stage of developing the Dr. Meta metaverse platform, we needed to consider regional disparities in medical resources and information and communication technologies to facilitate a multicenter cancer education environment using metaverse technology. However, we conducted research to demonstrate an example where multiple medical institutions could more actively use the metaverse by overcoming such physical constraints. We have established the Dr. Meta metaverse platform’s infrastructure and maintained its setting to disseminate and implement it in local communities. We aim to construct a more robust remote cancer education network with each RCC and other hospitals in multiple regions of the Republic of Korea by using the Metaverse Educational Center subplatform. In addition, we plan to develop an English version of Dr. Meta and intend to promote collaboration with foreign institutions. We anticipate that these future research directions will contribute to enhancing the generalizability of our study findings.

### Limitations

Although this study presented positive assessments of the cancer education metaverse platform and outlined prospects for its future development, several limitations exist. First, our results may be specific to a particular context, topic, or population, making it challenging to generalize the results to the overall level of education through a medical metaverse. In this study, we used the subplatform only for the remote mammography positioning training of health professionals. Hence, it is difficult to anticipate whether contextual differences affect our results. For instance, if the purpose of cancer education is not to train health professionals but to teach cancer care methods to patients or cancer prevention methods to the general public, the factors associated with intention for continuous use might differ. Demographic information, such as sex and age distribution ratios, might also vary, potentially affecting research outcomes. Further studies are needed to confirm whether the results of this study can be reproduced in the general population or other specific population groups.

Second, as this study used cross-sectional data, not all observed relationships among variables are actual causalities but associations; therefore, careful interpretation of causal assumptions is necessary. Although mixed methods can supplement explanations of the possibilities of some causal relationships, some aspects still need to be addressed. In particular, with the current research design, it is difficult to provide valid reasons for the relationships showing inconsistent results compared with other existing studies, such as those regarding interest or perceived ease of use. To explain the relationships among factors and their mechanisms concretely, an experimental study or other study using different research methods capable of providing evidence of causality should follow.

Third, because this study mainly tested the usability of the cancer education metaverse platform, its effects remain unknown. By expanding the scope of research to encompass variables related to the educational effectiveness of the subplatform, an inclusive understanding of its impacts can be achieved. However, developing a topic-focused questionnaire rather than a universal usability questionnaire is required to accomplish this goal. Efforts to develop survey methods that can overcome physical constraints and can be easily used by each cancer center and to explore a systematically validated questionnaire suitable for educational topics are required. We will consider conducting this subplatform effects study by seeking research methods specialized in a target content topic for metaverse cancer education.

### Conclusions

This study introduced a nationwide project in Korea using the Metaverse Educational Center subplatform of the Dr. Meta multidomain metaverse cancer care digital platform. The results of this study demonstrated the positive user experience of the subplatform. This study also identified variables that may influence the intention to use the subplatform continuously. We comprehensively described how to make effective plans for improving the user experience with the subplatform by considering how to enhance the evaluation of these variables. Although this study showing the subplatform’s positive user experience may fall short of demonstrating the full educational effectiveness of the subplatform, it may serve as evidence to expand the use of the subplatform and a catalyst for conducting future effectiveness research. Using this study as a starting point, if future research verifies the efficacy of the subplatform, it will be possible to argue the potential of the subplatform for successful remote cancer education more significantly. Moving forward, beyond mammography positioning training for health professionals, expanding the scope of this cancer education metaverse platform to include various topics and target populations may enable the establishment of a valuable system for successfully conducting remote cancer education. When operating educational intervention programs for at-risk patient populations with limited access to medical information or difficulty in hospital visits, the subplatform may play a key role in sharing knowledge. Moreover, it may provide a communication space that offers enjoyment and psychological support to younger patients who are relatively familiar with virtual spaces and new media (eg, adolescent and young adult patients). To bolster metaverse cancer education programs and amplify their effects in the future, it is necessary to explore the ways to secure their various contents and establish systems to promote their broad use.

## Appendix

Multimedia Appendix 1 The questionnaire used to evaluate participants’ experiences using Metaverse Educational Center.

## Abbreviations

AI: artificial intelligence
NCC: National Cancer Center
RCC: regional cancer center
TAM: technology acceptance model
VR: virtual reality

## Data Availability

The data sets generated or analyzed in this study are available from the corresponding author on reasonable request.
