# Supplementary material for: Factors Associated With Continuous Use of a Cancer Education Metaverse Platform: Mixed Methods Study
Source: J Med Internet Res. 2024 Jul 15;26:e57762. doi: 10.2196/57762 (PMC11287095; doi:10.2196/57762)
Supplement: Multimedia Appendix 1 [file jmir_v26i1e57762_app1.pdf]

Metaverse Educational Center User Experience Test

We are investigating the user experience of Metaverse Educational Center (one of the subplatforms in the Dr. Meta multidomain metaverse cancer care digital platform) to provide a better quality subplatform environment and services to those who have used the subplatform. Please give us your honest evaluation and opinions on the subplatform.

1. Consent to Participate in the Program & Consent to Collection and Use of Personal Information

1) I agree to participate in the program voluntarily. ☐ I agree ☐ I do NOT agree

2) I agree to the collection and use of my personal information. ☐ I agree ☐ I do NOT agree

2. Participation Information

1) Organization: _______________ 2) Date of Use: _______________

3. Basic Information

1) Sex: ☐ Male ☐ Female 2) Age: _____________

3) Position in the Hospital:

☐ Health professionals (doctors) ☐ Health professionals (nurses)

☐ Health professionals (other: radiologic technologists, physical therapists, pharmacists, etc.)

☐ Patients ☐ Caregivers

☐ Other (hospital administrative staff, educators, social workers, researchers, etc.)

4. Questions are asking about your thoughts about the Metaverse Educational Center subplatform after using it. Please indicate how much you agree with the statement below.

No.	Items	Response			
		Strongly Disagree	Disagree	Agree	Strongly Agree
Individual differences					
1	I am usually interested in using new technologies or devices.	①	②	③	④
2	I tend to not experience dizziness or motion sickness easily.	①	②	③	④
User experiences					
3	I was generally satisfied with using this subplatform.	①	②	③	④
4	This subplatform made me feel interested.	①	②	③	④
5	This subplatform made me feel immersed.	①	②	③	④
6	This subplatform was easy to use.	①	②	③	④
7	There was no discomfort (eg, dizziness and nausea) in using this subplatform.	①	②	③	④
8	There was no difficulty in wearing this subplatform device (ie, head-mounted display and controllers).	①	②	③	④
9	There was no difficulty in operating this subplatform device (ie, head-mounted display and controllers).	①	②	③	④

10	I want to continue using this subplatform in the future.	①	②	③	④

5. If you have any suggestions or improvements, please write them in.

Thank you for participating in this survey.
